# Regulation of BCR-dependent germinal center B-cell formation by HGAL and insight into its emerging myeloid ortholog, C1ORF150

**DOI:** 10.3389/fimmu.2024.1437516

**Published:** 2024-10-15

**Authors:** Paul Toran, Anthony Novelli, Jennifer Lazor, Alexandra Vachon, Don M. Wojchowski

**Affiliations:** ^1^ Department of Molecular, Cellular and Biomedical Sciences University of New Hampshire, Durham, NH, United States; ^2^ Biotherapeutics, Boehringer Ingelheim Pharmaceuticals, Inc., Ridgefield, CT, United States

**Keywords:** HGAL, C1ORF150, adaptor proteins, B-cells, mast cells

## Abstract

The specificity of cytokine and immunoreceptor signaling frequently depends upon receptor recruitment of select adaptor proteins and specifically engaged effectors. This review focuses on the orthologous adaptor proteins, HGAL and C1ORF150, and aims to provide insight into their respective modulation of lymphoid and myeloid cell signaling, formation, and function. HGAL acts predominantly within germinal center B cells as an important BCR signal transducer. Effects on BCR signalosome assembly involve HGAL’s localization to the plasma membrane via its lipidation, initial interactions with SYK, the pY-phosphorylation of HGAL including its recruitment of GRB2, and HGAL engagement of PDZ-RhoGEF and RhoA signaling. At ligated BCRs, this includes HGAL(−GRB2) stimulation of SYK kinase, attenuation of calcium flux-dependent and NF-κB expression, promotion of cSMAC formation, and cytoskeletal remodeling associated with HGAL-attenuated cell migration. HGAL and partnered effectors also impact on DLBCL pathogenesis, and studies are summarized on HGAL’s actions (using DLBCL and Burkitt lymphoma B cells) including cell migration effects, HGAL modulation of cytoskeletal components, and insightful HGAL transgenic mouse and xenograft models. For C1ORF150, its HGAL-homologous subdomains are considered, together with studies that demonstrate C1OR150’s FcϵRI- and KIT-mediated expression and phosphorylation in primary human mast cells. Intriguingly, recent GWAS studies have identified a *C1ORF150* in-frame splice variant that is strongly associated with urticaria. Candidate mechanisms via which the encoded “C1ORF150-Δexon2” isoform affects mast cell degranulation are considered, including FcϵR1 and/or KIT receptor connections, and candidate “myristoylation switch” mechanisms.

## 1 Introduction to HGAL and C1ORF150

For this review, one goal is to summarize progress in understanding key effects exerted by HGAL, an important B-cell receptor (BCR) adaptor protein within germinal center (GC) B cells (1–11). This includes HGAL’s diverse cellular effects (and their underlying molecular mechanisms) within three key response pathways: (a) HGAL’s differential effects on BCR-activated upstream signaling events (3, 7, 8, 12, 13), enhanced BCR clustering (7, 8), and supramolecular activation cluster (SMAC) formation (8, 10, 14); (b) mechanisms via which HGAL promotes cytoskeletal remodeling, in part through PDZ-RhoGEF recruitment (4, 5, 8, 10, 11, 15, 16); and (c) HGAL as a marker of diffuse large B-cell lymphoma (DLBCL) disease severity (1, 2, 17), and lymphomagenic driver (10, 13, 18).

Recently, a novel myeloid ortholog of HGAL, C1ORF150, has been described (19–26), which is conserved in *Homo sapiens* and primates but is not represented among mouse, rat, and lower vertebrate genomes. For C1ORF150, the goals of this review are as follows: (a) summarize C1ORF150’s primary structural features, including informative comparisons to HGAL; (b) define what has been observed to date regarding C1ORF150’s regulated expression (19, 21) and phospho-modifications in myelo-erythroid progenitors (22) and mast cells (24); and (c) highlight apparent roles of C1ORF150 during mast cell activation as implicated via GWAS associations of a *C1ORF150* splice variant with urticaria (23, 25, 26). Prospective studies additionally are suggested for HGAL and C1ORF150 towards extending insight into their cellular effects, molecular action mechanisms, and respective impact on lymphomagenesis and mast cell dysregulation.

## 2 HGAL’s subdomains and interactive motifs

**Figure 1:**
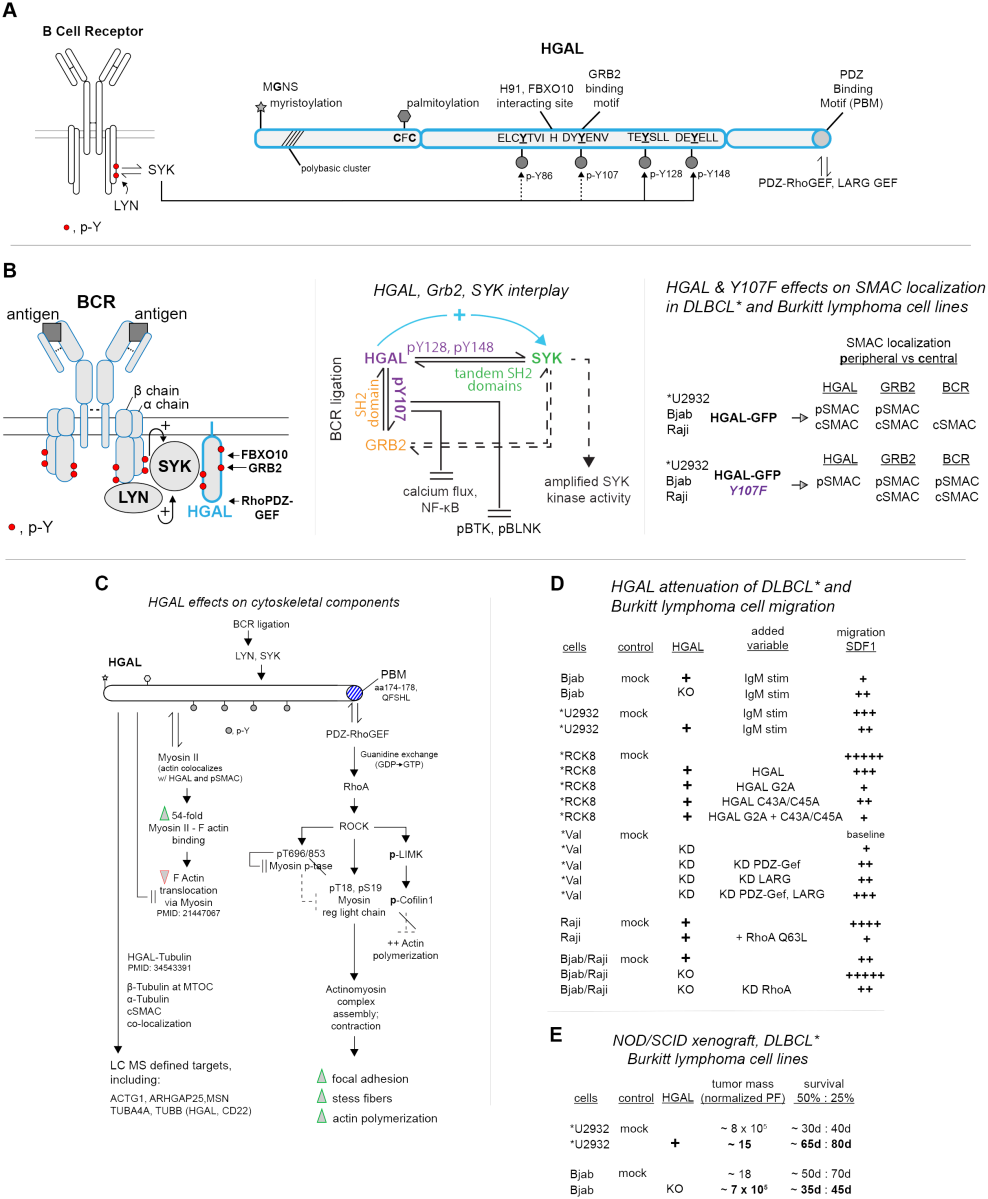
HGAL’s interactive domains and signaling partners within ligated BCRs, effects on cytoskeletal remodeling, and DLBCL and Burkitt lymphoma cell migration. **(A)** HGAL’s multi-fold interactive domains*—*upon BCR ligation within GC B cells, HGAL provides five sets of interactive motifs. These include N-terminal myristoylation and palmitoylation sites; four modulated central phospho-tyrosine (pY) motifs (with BCR-activated SYK as an indicated phosphorylating kinase); a validated DDpY107ENV binding site for GRB2 docking; a validated C-terminal PDZ-RhoGEF binding motif (PBM); and an H91 site demonstrated to bind FBXO10, (and associated Ub ligase). **(B)** HGAL, GRB2, and SYK interactions, and effects on BCR-upstream signaling and supramolecular activation complexes (pSMACs, cSMACs)—following BCR ligation, HGAL, as complexed with GRB2 and SYK has been demonstrated in *DLBCL and Burkitt lymphoma cell lines to activate SYK (left panel) while attenuating calcium flux and NF-kB activation and the actions of (p)BTK and (p)BLNK (center panel) (note, “*” designates DLBCL cell lines). These latter effects also depend upon HGAL’s pY107 GRB2 binding site. HGAL additionally supports the formation of peripheral and central pSMACs and cSMACs, with roles for HGAL-pY107 defined especially for the coalescing of BCR’s within cSMACs (right panel). **(C)** HGAL-mediated engagement of BCR-activated PDZ-RhoGEF pathways to DLBCL and Burkitt lymphoma cell cytoskeletal remodeling, including direct HGAL–cytoskeletal interactions. Right column: roles for HGAL’s C-terminal PDZ-RhoGEF binding domain are outlined in a propagating Rho/ROCK pathway to cytoskeletal remodeling. This includes the reinforcement of such signaling events due to increased levels of HGAL in DLBCL and Burkitt lymphoma cells (for details, see *Section 5*). Left column: HGAL additionally has been demonstrated to interact directly with select cytoskeletal components and to promote the formation of focal adhesions and stress fibers. **(D)** HGAL attenuation of DLBCL and Burkitt lymphoma cell migration (as mediated via a PDZ-RhoGEF, RhoA Rock pathway)—studies of DLBCL and Burkitt lymphoma cell line migration (in response to SDF1) are summarized. Variables are indexed as HGAL expression (+); HGAL knockdown (KD); IgM ligation of BCRs; and expressed HGAL constructs. Relative migration observed (migration chambers) is represented as +, ++, +++, ++++, +++++ (right column). **(E)** HGAL promotes DLBCL and Burkitt lymphoma cell line tumorigenesis—*in vivo* outcomes of xenograph studies (NOD/SCID mice) are summarized. DLBCL lines employed are *U2934 (HGAL negative, +/− HGAL ectopic expression) and Bjab (HGAL positive, +/− HGAL knockdown). Tumor mass and survival at d40 and d80 are summarized.

In considering HGAL directly, it is first noted that HGAL’s expression is restricted predominantly to GC B cells (1, 2). In addition, human germinal center-associated lymphoma (HGAL) is alternatively designated as GCET2 (3, 18), GCSAM, and “M17” (mouse) (27). Via concerted investigations of HGAL (using predominantly DLBCL and Burkitt lymphoma cell lines) (3, 5, 7–9, 15, 28–31), functional roles have been delineated for five substructural components (**Figure 1A**): (a) an N-terminal myristoylation site (MG2NS) (3, 7, 28, 31); (b) a proximal palmitoylation site (C43FC45) (3, 7, 28); (c) four central phospho-tyrosine (pY) sites, including a consensus GRB2 binding site (pY107ENV) (3, 8); (d) a defined C-terminal PDZ binding motif (Q174FSHL) that recruits PDZ-RhoGEF (5, 15, 32); and (e) a His91 site that binds FBXO10, a substrate recognition component of SCF E3 ubiquitin ligase complexes (29, 30) as a driver of HGAL degradation (9). In addition, a “polybasic cluster” (Arg-7, Arg-10, Arg-11, and Arg-21) (33, 34) adjacent to HGAL’s myristoylation site is defined.

HGAL’s lipidation sites each have been defined (via [^3^H]-myristic and palmitic acid labeling and mutation) to mediate HGAL’s plasma membrane localization (3, 7, 28, 31). The sequences and spacing of these sites (together with HGAL’s adjacent “polybasic cluster”) are consistent with a “myristoyl–palmitoyl switch” that can dynamically modulate plasma membrane protein localization (35, 36).

For HGAL’s phospho-tyrosine (pY) motifs (8), the sequence of “pY site-2” (pY107ENV) is a consensus SH2-domain binding site (37). Via pY->F site mutation, co-expression, and co-immunoprecipitation studies, GRB2 has been demonstrated to bind at HGAL-pY107 (3, 8). HGAL’s pY128 and pY148 sites approximate ITAM motifs (8, 13, 38, 39), and co-immunoprecipitation experiments using epitope-tagged HGAL truncation and pY->F constructs (expressed together with SYK in DLBCL VAL cells) have demonstrated SYK binding to HGAL via HGAL’s paired C-terminal pY motifs (13). Singular Y128F or Y148F mutations, however, do not interrupt HGAL-SYK co-immunoprecipitation (13). HGAL’s C-terminal consensus Q174FSHL PDZ protein binding motif (PBM) (X-S/T-X-Φ) (15, 32) has been shown (via co-IP experiments using HGAL PDZ-RhoGEF domain deletion constructs) to specifically bind the Dbl region of PDZ-RhoGEF (5).

The above studies demonstrate that, despite its 21 kDa size, HGAL can dynamically interact via five sets of sub-domains to mediate plasma membrane localization and engage signaling effectors that co-regulate BCR response pathways.

## 3 Roles for HGAL upon BCR activation

This section seeks to summarize studies that connect HGAL’s interactive motifs to BCR-activated upstream signaling pathways. HGAL was discovered in DLBCL patients as a marker of disease severity and overall survival (OS) (1, 40) (with DLBCL comprising ~35% of non-Hodgkins lymphomas). This, and novel HGAL connections to cytokine signaling (1), broadened interest in HGAL’s actions. In a *M17/Hgal* knockout mouse model, B- or T-cell populations were not altered (although numbers of Peyer’s patches were lessened) (41). Class type switching and NPCG-induced memory B-cell formation also were largely unaffected (41). This redirected investigations to HGAL signaling in human GC, and DLBCL or Burkitt lymphoma cell lines. (Note: In this report, DLBCL* lines are denoted by an asterisk “*”).

Upon ligation, BCR upstream signaling (**Figure 1B**, left panel) is initiated via LYN phosphorylation of ITAMs within BCR alpha and beta chains (42, 43). These pY sites then become docking sites for SYK kinase. This docking (and LYN phosphorylation of SYK Y348 and Y352 sites) leads to SYK activation (44). HGAL’s phosphorylation by SYK has been demonstrated *in vitro* using recombinant TRX-HGAL and SYK, with SYK phosphorylating HGAL at pY86/107/128 sites, and pY80 (8). For HGAL-Y148, IL6 can selectively induce its phosphorylation (4). For HGAL-pY107, this site has been shown to mediate GRB2 binding, as validated through GST-GRB2 pull-down assays using TRX-HGAL and Y107->F forms (+/− SYK or LYN as phosphorylating kinases). Additionally, SYK knockdown decreased co-IP of HGAL with GRB2 (8). In Raji cells, BCR ligation enhanced HGAL’s co-IP with SYK while decreasing the co-IP of GRB2 and SYK (8). The knockout of HGAL did not affect SYK and GRB2 co-IP’s. GRB2-KO, however, decreased HGAL’s co-IP with SYK (in BCR-ligated cells, but not unstimulated cells) (8). In kinase assays using SYK immunoprecipitated from IgM-challenged Raji cells, HGAL enhanced SYK activity (8, 13), while GRB2 was without effect (8). In assays using SYK and HGAL, the addition of GRB2 did not diminish HGAL-enhanced SYK activity. These findings indicate roles for SYK in mediating HGAL-GRB2 interactions, together with GRB2-mediation of HGAL–SYK interactions. Therefore SYK kinase activation can be regulated by its singular and/or combined interactions with HGAL and GRB2 (**Figure 1B**, center panel).

To assess HGAL’s signaling effects following BCR-ligation, DLBCL and Burkitt lymphoma cell lines have been used (together with HGAL mutated at its GRB2 binding site, pY107->F). In IgM-stimulated Raji cells, HGAL-KO decreased calcium flux, while GRB2-KO enhanced calcium flux (8). Reconstitution of HGAL-KO cells with HGAL-GFP or HGAL-Y107->F each restored calcium flux, with HGAL-Y107->F yielding calcium flux comparable to unmodified cells. In BCR-stimulated *U2932 cells, HGAL-Y107->F (vs. mock and WT-HGAL) expression yielded greater increases in calcium flux, NF-kB transcriptional reporter activity, and phosphorylation of SYK, BLNK, and BTK (8). These studies suggest that HGAL (and GRB2) moderate BCR-dependent calcium flux and NF-kB expression and the phosphorylation of BLNK and BTK (as drivers of early B-cell development) (45, 46). Via select partnered effectors, HGAL therefore can up-modulate SYK signaling while attenuating other BCR-triggered upstream signaling pathways.

Expression of HGAL (GFP-fusion), and of HGAL-GFP-Y107F, also differentially affected BCR-induced SMAC formation (8). In DLBCL and Burkitt lymphoma cell lines (with endogenous HGAL KO’s) wt-HGAL expression reinforced dense cSMAC formation [induced by anti-human IgM (FAB′)2] with HGAL co-localizing with BCR’s and GRB2. Within pSMACS, HGAL additionally co-localized with actin. In contrast, for HGAL-Y107->F, this GRB2 binding-deficient HGAL construct localized to pSMAC domains, with BCR’s and GRB2 localizing to relatively dispersed cSMACs (**Figure 1B**, right panel). This points to further interactive effects of HGAL-GRB2 in this coalesced BCR context. Interestingly, SMACs in T-helper cells (14, 47, 48) also include HGAL (and SYK), and HGAL recently has been described as a marker of follicular T-helper cell lymphoma (49).

## 4 HGAL-mediated cytoskeletal restructuring and attenuation of cell motility

A series of investigations have first delineated an HGAL-dependent pathway via which HGAL’s consensus PBM motif (15, 32) recruits PDZ-RhoGEF, heightens RhoA activity, and leads to cytoskeletal component restructuring in DLBCL and Burkitt lymphoma cell lines (4, 5, 8, 15, 16, 32, 50–55) (see **Figure 1C**, right column). Specifically, the siRNA knockdown of HGAL in Raji and *VAL cells decreased levels of GTP-bound RhoA (5), as did miR-155 (that targets HGAL’s 3′ UTR) (11). HGAL’s knockdown additionally decreases Rho-kinase (ROCK) activity and consequently ROCK’s phosphorylation of myosin regulatory light chain (MRLC), myosin phosphatase subunit MYPT1, and cofilin (5). Phosphorylation of MRLC (S19, T18) promotes actomyosin complex assembly, contraction, and filament assembly (54). ROCK’s phosphorylation of MYPT1 is inhibitory and sustains MRLC activity (50, 51). ROCK further phosphorylates LIMK, which phosphorylates cofilin to inhibit cofilin’s effects on actin disassembly (52, 53). Additionally, HGAL may activate RhoA via PI3K downstream of HGAL’s BCR-dependent SYK activation (56). These BCR- and HGAL-induced RhoA and ROCK signaling events promote F-actin polymerization and heighten focal adhesion and stress fiber formation (determined via flow cytometry, immunofluorescence, and interference-reflection contrast microscopy) (5).

A parallel path characterized for HGAL’s effects on cytoskeletal components (**Figure 1C**, left column) first involves HGAL’s co-localization with actin (4, 8, 16), myosin II (4), and tubulin (55) as observed in HeLa, *SUDHL6, *VAL, and Raji cells via immunofluorescent confocal microscopy. Second, evidence of direct interactions of HGAL with these, and ~30 additional cytoskeletal proteins, has been provided via MS analysis of affinity-purified proximity-biotinylated HGAL interactors (55). Co-sedimentation and co-IP experiments also demonstrate HGAL’s association with actin, myosin (16), and tubulin (55). HGAL additionally can enhance myosin II and F-actin interactions (16). Such effects implicate HGAL regulation of DLBCL B-cell migration and/or adhesion, with potential effects on dissemination.

Evidence for HGAL attenuation of DLBCL and Burkitt lymphoma cell lines migration has been provided in studies using *U2932, *Val, *RCK8, Bjab, and/or Raji cells (5, 55, 57) (**Figure 1D**). In chamber migration assays (using fibronectin), endogenous and ectopic expression of HGAL in IgM-stimulated *U2932 or Bjab cells (respectively) significantly attenuated SDF1-induced cell migration (55). Studies using *RCK8 cells further demonstrated attenuation of migration as also enforced by an HGAL palmitoylation mutant (C43A/C45A) and more so by an expressed HGAL myristoylation mutant (MG2A) (7) (indicating that these attenuating effects of HGAL may occur away from the plasma membrane). In *VAL cells, the combined knockdown of HGAL, LARG, and PDZ-RhoGEF markedly reversed attenuated migration, as did individual HGAL and RhoGEF knockdowns (to a lesser extent) (5). In addition, ectopic expression of constitutively active RhoA-Q63L in Raji cells attenuated SDF1-dependent migration (55). Unexpectedly, the knockdown of RhoA also attenuated SDF1-dependent cell migration (regardless of HGAL-knockout) (55). These latter findings suggest that HGAL’s attenuation of cell migration might not depend strictly upon a RhoA pathway. These cell migration studies indicate that BCR and HGAL engagement of a PDZ-RhoA pathway can substantially attenuate DLBCL B-cell migration. Possible effects on cell adhesion (and contributing proteins) will also be meaningful to investigate, together with assessments of primary human DLBCL B-cell migration using B cells from patients with high vs. moderate HGAL expression levels.

## 5 HGAL’s regulated expression and actions as a driver of DLBCL

The discovery of HGAL as a DLBCL marker (1, 2, 10, 17) and disease severity index (OS) (6, 58, 59) prompted studies of *HGAL’s* expression profiles and gene regulation. Beyond GC B cells, *HGAL* (at the transcript level) is also expressed in naive and switched B cells, naive and memory CD4 T cells, and effector CD8 T cells (https://proteinatlas.org; https://fantom.gsc.riken.jp/5/). *HGAL’s* up-modulated expression was first observed upon IL4 activation of its JAK1/3, TYK2 coupled receptor in human peripheral blood lymphocytes (1), and upon IL13- activation of its JAK2, TYK2 coupled receptor (17). These effects suggest HGAL’s possible induction during inflammation (60–62). For HGAL’s down-modulation, one contributing factor is PRDM1 (“PR domain zinc finger protein 1”). Knockout studies using mouse models have defined PRDM1 as a prime regulator of B-cell transitions to plasma cells (63). As a clinical connection, clonal inactivating mutations of PRDM1 are associated with DLBCL (58, 64). Furthermore, PRDM1 represses *HGAL* expression at its proximal promoter at nt 1603 and 1383 sites (*Val and Raji cells) (6). Additionally, increases in HGAL due to PRDM1 mutation may drive DLBCL.

In early studies, transgenic mice expressing HGAL from a Sca1-promoter demonstrated effects on polyclonal B-cell lymphoid hyperplasia, hypergammaglobinuria, and amyloid-A amyloidosis (13). Observed increases in Syk phosphorylation, B-cell proliferation *ex vivo*, and RhoA activity each further indicated escalated BCR activation. Recently, studies using Cre-activatable Rosa26-HGAL transgenic mouse models have provided important evidence for HGAL DLBCL driver effects (10). Here, following the induced expression of HGAL in HSC (Sca1-Cre), PB proB cells (Mb1-Cre), or GC B cells (Aid-Cre) each promoted DLBCL-like lymphomas. Blocks in B-cell development at a GC-reactive stage were additionally observed. Effects on candidate DLBCL co-driver gene expression were also defined, including *Rgs1*, an inhibitor of lymphocyte migration (60), and GTPase *Rab10*, which (like HGAL) is elevated in DLBCL patients with extended OS (61). Mutations in additional genes implicated as secondary DLBCL drivers (62) were further defined, with a GC-B DLBCL lymphoma sub-type indicated in these important new mouse models.

To assess effects of HGAL on DLBCL tumorigenesis, human DLBCL and Burkitt lymphoma cell lines have been assessed in xenograft studies (NOD/SCID mice) (10). Cells included HGAL-negative *U2932 cells with (vs. without) ectopically expressed HGAL, and HGAL-positive Bjab cells with (vs. without) *HGAL* knockout. For *U2932 DLBCL cells, HGAL expression decreased tumor mass and increased survival relative to *U2932 controls (**Figure 1E**). In Bjab cells, HGAL’s knockout increased tumor mass while limiting overall survival. *In vivo* evidence therefore is provided for HGAL’s attenuation of DLBCL B-cell lymphomagenesis. The extent to which these outcomes may involve strengthened adhesion will also be informative to establish.

In mouse models, increased HGAL expression (within HSC, PB proB, or GC B cells) can contribute causally to onset of DLBCL-like disease. In xenograft models (using *U2932 and Bjab cells), however, HGAL expression can reinforce a PDZ-RhoGEF signaling pathway that attenuates migration and slows tumorigenesis. Similarly, among DLBCL patients with elevated HGAL, relatively high HGAL levels correlate with improved OS. HGAL, therefore, is paradoxically indicated to be not only a DLBCL driver but also, at elevated levels, a favorable prognostic indicator. For these patients, HGAL’s effect on attenuated mobility may, in part, underly improved OS. For this apparent contradiction, effects of distinct GC-B DLBCL driver mutations might also be involved.

## 6 C1ORF150 as a novel ortholog of HGAL with implicated roles in cytokine and immunoreceptor signaling, and urticaria

### 6.1 C1ORF150’s discovery, expression profiles, and structural comparisons with HGAL

C1ORF150 was first discovered as a target of EPO/EPOR/JAK2-induced pY-phosphorylation in myelo-erythroblastic UT7epo cells (22). Unlike *HGAL*, *C1ORF150* is expressed predominantly in HSC, MPP, CMP [also AML inv (16)/t(16:16) and t(15:17) leukemia] (www.bloodspot.eu), and maximally in mast cells (19, 23, 25, 26). Among primates, *C1ORF150* is highly conserved but is absent among mouse, rat, and lower vertebrate genomes (22).

**Figure 2:**
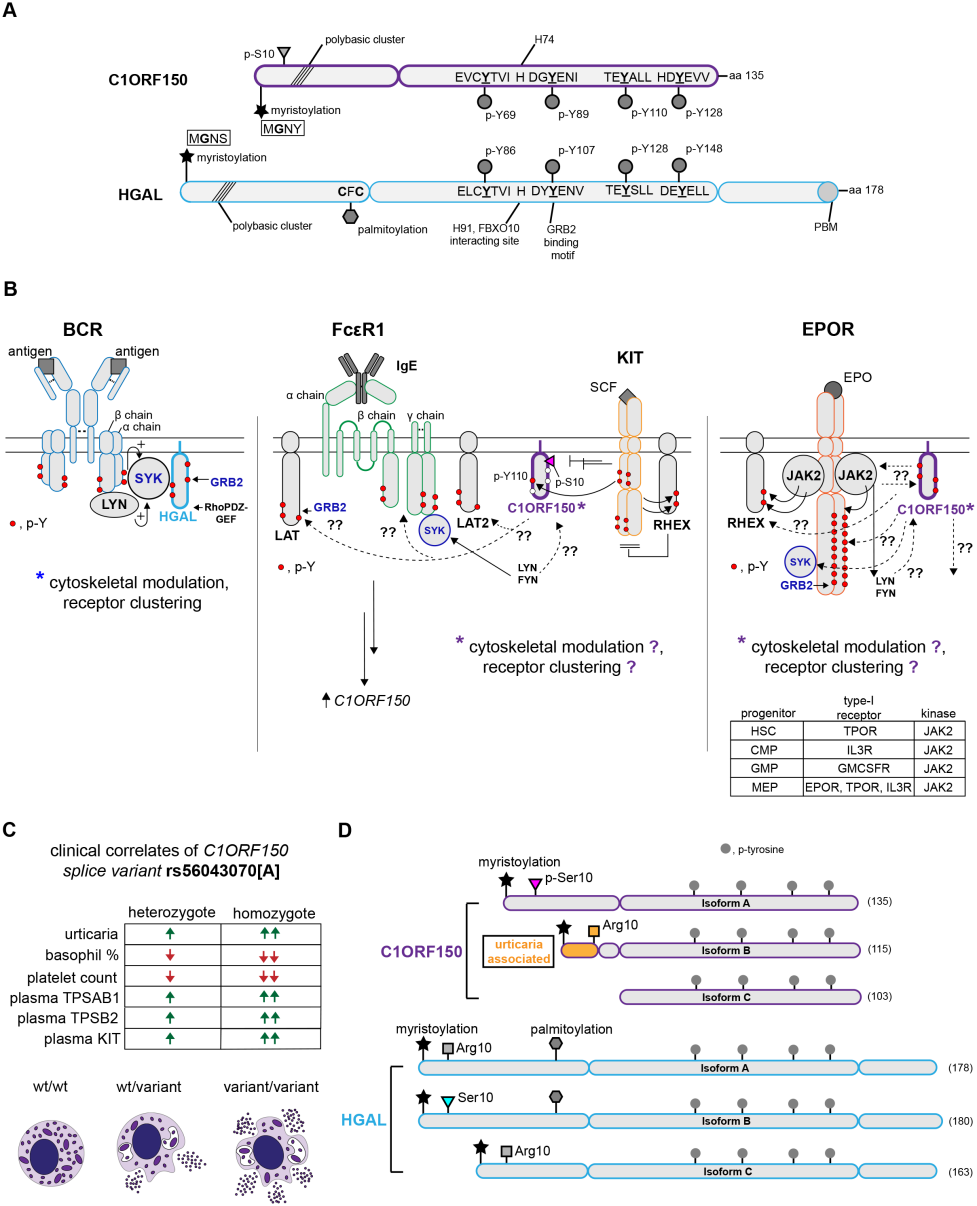
C1ORF150 and HGAL homology; prospective roles of C1ORF150 as an adaptor protein within FcϵR1, KIT, and EPOR/JAK2 complexes, and GWAS association of a C1ORF150 splice variant with urticaria. **(A)** hC1ORF150 and h-HGAL conserved motifs include C1ORF150’s N-terminal MG2NY predicted myristoylation site and four pY-modulated and similarly spaced sites. A shared consensus GRB2 binding site [GY89EN(I)] and tandem C-terminal pY sites in C1ORF150 share similar sequences (and relative positions) as HGAL’s pY128 and pY148 sites (as SYK docking sites in HGAL). Unlike HGAL, C1ORF150 also lacks any candidate site for palmitoylation or PDZ-RhoGEF binding. **(B)** Models outlining prospective roles for C1ORF150 within FcϵR1, KIT, and hEPOR/JAK2 membrane–proximal signaling complexes. Left panel: as a comparator, core components of the BCR are diagrammed, including the LYN, BCR alpha, beta chain, and SYK-mediated recruitment of HGAL, together with GRB2. Center panel: the mast cell FcϵR1 is diagrammed, together with KIT as a positive co-regulating factor for mast cell development, and activation. IgE/FcϵR1 induction of *C1ORF150* transcript expression is also diagrammed. SYK and GRB2 as FcϵR1 signal transducers also are outlined. For KIT, SCF-induced phosphorylation of C1ORF150 at pY110 together with an induced de-phosphorylation at (p)Ser10 is also diagrammed. KIT-mediated pY modulation of RHEX is also depicted. Right panel: based on the EPO/EPOR-mediated pY phosphorylation of C1ORF150, its plasma membrane localization, and its HGAL-homologous motifs, an initial basic model is framed for C1ORF150’s coupling with EPOR/JAK2 signaling complexes. Type-1 JAK2-coupled receptors (lower box insert) among hematopoietic progenitor cells with elevated *C1ORF150* expression also are indicated. **(C)** For the urticaria GWAS-associated *C1ORF150* splice variant, rs56043070[A], clinical correlates in heterozygous and homozygous patients are summarized, and splice variant associated heightened activation and degranulation are diagrammed. **(D)** Isoforms of C1ORF150, and HGAL (translated from validated transcripts) including urticaria associated *C1ORF150* “isoform B”. Upper panel: for C1ORF150, its urticaria-associated transcript-encoded protein is diagrammed including its predicted 20-residue deletion (encoded by exon-2). For this variant transcript, C1ORF150’s predicted myristoylation site is retained but with the conversion of an adjacent (p)Ser10 residue to Arg10. Lower panel: for HGAL, in isoform B, Arg10 interestingly becomes converted to Ser10. In Isoform C, HGAL’s palmitoylation site is deleted, with Arg 10 represented (rather than S10).

Within C1ORF150, substantial homology exists between, first, HGAL’s validated myristoylation site with MG2NY in C1ORF150 (**Figure 2A**). Homology is also significant across four similarly spaced pY-motifs within C1ORF150, and HGAL (8, 20, 22). C1ORF150’s pY89EN site closely conforms to HGAL pY107EN (a confirmed GRB2 binding site) (3, 8, 37). In HGAL, C-terminal pY128 and pY148 sites can function as ITAM docking sites for SYK (8, 13, 38, 39), and C1ORF150’s similarly positioned pY110, pY128 sites may likewise recruit SYK. For C1ORF150 (p)Y69 and corresponding HGAL-pY86 sites, interacting partners are undefined, but each are consensus ITIM’s (S/I/V/LxYxxI/V/L) with PTPs recruitment potentials (65, 66). C1ORF150 is distinct from HGAL first in lacking HGAL’s palmitoylation motif (MG2NY as C1ORF150’s singularly indicated lipidation and candidate plasma membrane localization motif) (20). In C1ORF150, a (p)Ser10 site (absent in HGAL) occurs proximal MG2NY. Notably, C1ORF150 additionally lacks HGAL’s C-terminal domain, including its PBM motif (5) (with no apparent C1ORF150 site for PDZ-RhoGEF engagement).

Together, C1ORF150’s expression profiles and structural features point to roles as a multi-site adaptor protein in HSC, MPP, CMP, and human mast cells. As considered below (*Section 6.2*), this extends to C1ORF150’s possible effects on signaling among KIT, FcϵR1, and/or type-1 JAK kinase-coupled receptors.

### 6.2 C1ORF150 connections to FcϵR1, KIT, and EPOR/JAK2 signaling

Within BCR complexes, insight into HGAL’s recruitment and signaling capacities (outlined in **Figure 2**, left panel) provides an initial framework for considering ways via which C1ORF150 might function within FcϵR1, KIT, and EPOR/JAK2 signalosomes.

For FcϵR1, connections to C1ORF150 are presently twofold. Within primary human dermal mast cells, FcϵR1 ligation induces rapid increases in *C1ORF150* transcripts (19) (**Figure 2B**, center panel). GWAS-defined dysregulation of mast cells as associated with splice variant (rs56043070A) encoding C1ORF150-Δexon2 (25, 26) further is consistent with a role of C1ORF150 within a degranulation activation pathway.

In mast cells, KIT signaling can amplify FcϵR1-propagated signals for mast cell activation (67, 68). KIT ligation can rapidly induce C1ORF150’s phosphorylation at pY110 (24) (corresponding to pY128 in HGAL, an indicated SYK docking site) (**Figure 2B**, center panel). KIT ligation also induces C1ORF150’s rapid dephosphorylation at (p)Ser10 (24) (proximal to its myristoylation site polybasic cluster). Although discovered as an EPOR/JAK2 pY phosphorylation target (**Figure 2B**, right) beyond the commonality of roles for SYK and GRB2 in EPOR/JAK2 signaling, more work is needed to assess C1ORF150 as a proposed new component of the EPOR (and potentially additional JAK2-coupled type-1 hematopoietin receptors).

### 6.3 Implicated effects of C1ORF150 on mast cell activation

Urticaria is diagnosed by a well-defined set of clinical markers (69). This aided the focus of meta-GWAS studies (25, 26) that discovered (3.6×10^−44^ association p-value) an urticaria-associated *C1ORF150* splice variant [rs56043070A] (C1ORF150-Δexon2) across four patient cohorts (25). Associations of [rs56043070A] (C1ORF150-Δexon2) were also defined for decreased % basophil and platelet counts (25). For the Icelandic cohort, elevated TPSAB1, TPSB2, and KIT markers were additionally associated with [rs56043070A] (C1ORF150-Δexon2) (25). Based on the above markers, homozygosity for [rs56043070A] (vs. heterozygosity) also conferred the above-additive (vs. expected) risk (25) (**Figure 2C**). *C1ORF150* splice variant [rs56043070A], C1ORF150-Δexon2 encodes an in-frame protein with an exon-2 encoded 20-residue deletion and a Ser10 to Arg10 substitution (**Figure 2D**, “isoform B”).

## 7 Concluding remarks: of mice, men, myristoylation, and mast cells

### 7.1 Mice and men

The establishment of cre-activatable Rosa26-HGAL mouse lines that give rise to DLBCL-like lymphomas with GC involvement provides new inroads for defining HGAL domains and coupled pathways that affect malignancies. For example, HGAL’s C-terminal PDZ-RhoGEF-binding motif (“PBM”) is proving to be important for GC DLBCL B-cell migration and dissemination (5, 55). The construction of mice expressing HGAL with a mutated PBM would provide *in vivo* insight into HGAL-mediated PDZ-RhoGEF signaling. Notably, within Hgal/M17, its candidate PBM (unlike h-HGAL’s PBM) is inconsistent in its sequence with the recent comprehensive delineation of PBM sequences across three classes and 16 subclasses (70). To avoid possible complications in interpreting HGAL effects in mice harboring endogenous murine Hgal/M17, *Hgal/M17* knockout mice could be used in which B- and T-cell formation and function are only nominally affected (41).

### 7.2 Myristoylation

Structurally, C1ORF150’s adjacent consensus N-terminal myristoylation site, (p)Ser10, and “polybasic cluster” (**Figure 2A**) meet all requirements of an electrostatic-myristoylation switch in which lipidation-dependent plasma membrane association is opposed by phospho-Ser10 (35, 36). In urticaria-associated C1ORF150-Δexon2, (p)Ser10 is replaced by Arg10, negating predicted electrostatic membrane repulsion (**Figure 2D**). By comparison, HGAL’s N-terminal myristoylation and palmitoylation sites predict a myristoyl–palmitoyl type switch (35) in which stepwise myristoylation and palmitoylation regulate plasma membrane localization (7). In addition, in HGAL isoform B, its polybasic cluster is disrupted by Ser10 replacing Arg10 (with Arg10 shifting to Arg12). For these homologous adaptors, the regulation of their plasma membrane localization by these predicted myristoyl switches merits investigation.

### 7.3 Mast cells

While C1ORF150 was discovered in a myelo-erythroid cell line as a target of EPOR/JAK2-induced pY-phosphorylation (22), this adaptor has been shown via LC-MS to be among the top 10 most abundant proteins in human primary dermal mast cells (21). Our laboratory additionally discovered C1ORF186/RHEX as a novel EPOR/JAK2-pY-modulated plasma membrane adaptor that also has been shown to be highly abundant in primary human mast cells (21). In these cells, KIT ligation further has been shown to modulate the phosphorylation of not only C1ORF150-pY110 but also RHEX-pY132, pY141 (24, 71). Furthermore, RHEX has been shown to inhibit KIT signaling (**Figure 2B**, center panel) (71). Notably, neither C1ORF150 nor RHEX is represented among mice, rats, or lower vertebrates. For C1ORF150 and RHEX, the extent to which these adaptors may contribute to increasingly realized differences between human and mouse mast cell signaling merits investigation [including differential FcϵR1 compositions (72) and mast cell subtypes (73)].
